# COVID‐19 vaccination, time for a second breath?

**DOI:** 10.15252/emmm.202215810

**Published:** 2022-02-25

**Authors:** Christiane E Gerke, Bernd Pulverer, Philippe J Sansonetti

**Affiliations:** ^1^ Institut Pasteur Université de Paris Innovation Office, Vaccine Programs Paris France; ^2^ EMBO Heidelberg Germany; ^3^ Institut Pasteur & Collège de France Paris France

## Abstract

One year into the Covid‐19 vaccination campaign, C. Gerke, B. Pulverer and P. Sansonetti discuss its results and redefine its objectives.

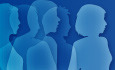

Approximately 1 year after the first cases of SARS‐CoV‐2 were reported in Wuhan, China, three major types of vaccines against the virus received market authorization—much faster than the previous record holder, the mumps vaccine, which took 4 years from development to deployment in the late 1960s. Of the more than 300 vaccine candidates against SARS‐Cov‐2 in development, 140 have so far reached clinical evaluation (https://www.who.int/publications/m/item/draft‐landscape‐of‐covid‐19‐candidate‐vaccines. accessed 24.01.2022); about 20 are currently in public use. These can be distinguished into five classes: classical inactivated virus vaccines “à la Salk”; recombinant adenovirus‐based (Ad) vaccines; mRNA vaccines; recombinant proteins; and DNA vaccines. Ad vaccines, such as those produced by AstraZeneca and Johnson & Johnson, represent the second successful viral vector‐based approach after the VSV‐based vaccine that terminated the Ebola epidemic in Western Africa in 2015. The two licensed mRNA vaccines developed by BioNTech/Pfizer and Moderna are first in class. Their unprecedented development in < 11 months from preclinical tests to approval has been made possible by the existing technology platform originally developed for cancer immunotherapy.

The recombinant Ad and mRNA vaccines also benefited from earlier research in response to the SARS‐CoV and MERS‐CoV outbreaks that had already narrowed the best vaccine antigen to the spike (S) protein. Both platforms represent a true technological breakthrough, on account of the increased speed of development and their versatility to adapt to novel genotypic variants showing antigenic switch during the course of an epidemic. Unlike inactivated vaccines, they also elicit significant levels of neutralizing anti‐S IgG, similar to or higher than those elicited by natural infection. First vaccinations with these two vaccine types provided strong population protection against infection and clinically severe outcomes in western countries, in line with prelicensure efficacy studies (Alderson *et al*, 2021). It consequently saved society from prolonged or repeated lock downs with their long‐term socioeconomical and psychological damage.

mRNA vaccines, however, suffer from one drawback: the necessity for frozen conservation, which makes their deployment difficult in low‐income countries without continuous cold chains. Recombinant adenovirus vaccines, which also sell at a far lower price, are thus likely better suited for these regions. Yet, other options need to be urgently considered as the vaccine coverage in low‐income countries remains at around 10% (Mathieu *et al*, 2022). The widening gap in global vaccine equity is ethically unacceptable and untenable from a public health point of view (Fig. 1).

**Figure 1:**
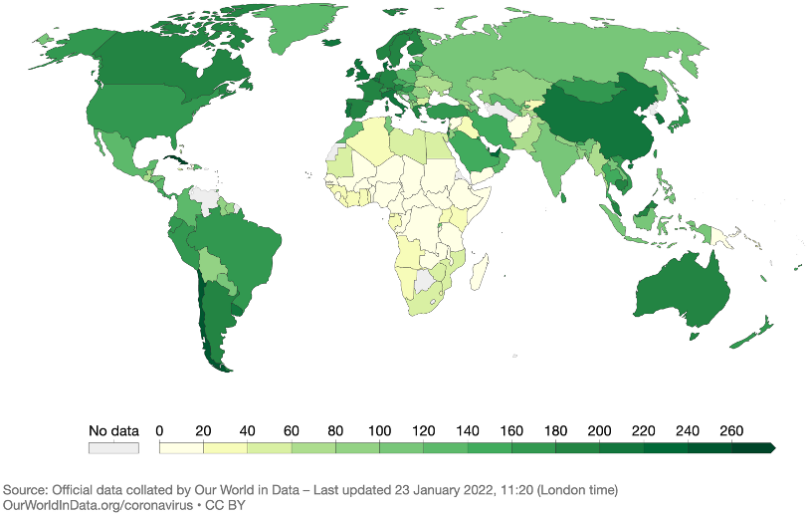
COVID‐19 vaccine doses administered by 100 people (22.01.2022) All doses, including boosters, are counted individually. Official data collected by Our World in Data (Mathieu *et al*, 2022).

After 1 year of intensive vaccination in western countries, we now see a spectacular drop of the number of patients with severe forms of COVID‐19, attested by the 1/9 average ratio of vaccinated vs nonvaccinated patients admitted to ICUs. Nonetheless, some weak points have emerged, which require adapting vaccine strategies in this second year as we face two new coinciding waves of the Delta and Omicron variants and subvariants. Even if the vaccines still protect against severe disease—at least for the time being—their effectiveness to prevent the circulation of Delta, and even more Omicron and its BA.2 sister variant, is decreasing (Eyre *et al*, 2022), although there are tentative signs that the waves have peaked in a number of countries.

Another drawback of the mRNA vaccines is the rather short duration of high IgG titers, probably contributing to the increasing occurrence of mild and moderate disease in vaccinated people and the reduced transmission blocking of the new variants (Collier *et al*, 2021). This might be due to the fact that mRNA vaccines do not optimally stimulate the balance between the maturation of IgG‐producing plasmablasts and memory B cells with the help of follicular T cells after antigen presentation in the germinal centers of draining lymph nodes. Clinical research will have to confirm this potential deficit in B cell memory and identify potential solutions.

The limitation of mRNA vaccines in blocking viral circulation is likely caused by a combination of factors. First, a weak stimulation of nasopharyngeal mucosal immunity, including poor, if any, production of neutralizing S protein‐specific secretory IgAs. Second, the T cell response, mostly via activated CD8+ T cells recognizing and killing cells undergoing viral replication, is weak and T cell memory accordingly limited in both the systemic and mucosal compartment (Collier *et al*, 2021). Systemic vaccination against invasive bacterial pathogens such as *Neisseria meningitidis*, *Haemophilus influenzae* b, and *Streptococcus pneumoniae* has shown that high serum IgG titers achieve significant neutralization of pathogen colonization following their transudation through the upper respiratory tract mucosa (Jochems *et al*, 2017). Thus, there is an urgent need to better understand the mucosal defenses following natural SARS‐CoV‐2 infection and vaccination. In addition, the progressive drift of the S protein between emergent variants reduces the immune protection to S protein‐based vaccines and may require sequential adaptation of the antigen.

In light of this constantly evolving situation, it seems important to reassess the actual objectives of the vaccination campaign and give it a “second breath.”

One can see two major strategies. The first is to strengthen the initial aim of “protection against the disease”, essentially by preventing severe forms without necessarily blocking the circulation of the virus. This would benefit at‐risk individuals, provide much needed relief for health institutions and their staff, and thereby lessen the dramatic impact of COVID‐19 on the care for other pathologies. In other words, we need “to learn to live with the virus” as is frequently proclaimed in Europe and North America, and accept an endemic situation, possibly with a seasonal profile similar to flu. In this setting, the increasing number of COVID‐19 treatments would help to curb the seasonal peak of disease, particularly in the most vulnerable and immune‐compromised patients. However, knowing the evolutionary versatility of this virus and the likely persistence of human and animal reservoirs, it would still allow the emergence of novel variants with unpredictable transmissibility and virulence. “Living with the virus” would necessitate rapid global vaccination, regular boosters with vaccines readjusted to excessive drifts of the S protein antigenicity, and strict surveillance of current and potentially new at‐risk populations, including children in whom current variants are intensively circulating.

The alternative trajectory is “disease elimination” by blocking viral circulation. It seems that reflection is warranted, as the current vaccination campaign remains largely reactive to the epidemiological situation. We would never dare, at this stage, to seriously consider viral eradication as was achieved with Smallpox. The performances of the current vaccines, the large percentage of asymptomatic infected people, the amazing efficiency of aerial transmissibility, and the potential existence of persistent animal reservoirs all defy any chance of eradication. Instead, we should consider the fight against measles as a model. With an *R*_0_ between 12 and 20—not dissimilar to Omicron—measles requires a 95% vaccination coverage to achieve herd immunity. Many other attributes of the virus, including efficient airway transmission, suggest that this is best achieved by a live‐attenuated, replicative vaccine that offers strong B and T cell response and memory (Gans *et al*, 2013). Indeed, the recent resurgence of measles in high‐income regions is the result of a slow erosion of vaccine coverage (Gastañaduy *et al*, 2021).

On this basis, how could we adjust our COVID‐19 vaccination tools and strategies to reach the same performance as measles vaccination, keeping in mind that the measles virus is genotypically more stable than SARS‐CoV‐2?

Improving B‐ and T‐cell memory is largely beyond the scope of the current vaccines and would need urgent fundamental research in vaccinology. On the other hand, implementing better control of virus circulation seems within reach: it would involve eliciting solid mucosal immunity to neutralize the virus at its entry site. This goal could be achieved in two ways. One approach is to combine systemic with mucosal vaccination. Several vaccine candidates for intranasal administration are currently in clinical trials (https://www.clinicaltrials.gov/ct2/results?cond=COVID‐19&term=intranasal+vaccine, accessed 22.01.2022), including a combined intranasal and intramuscular approach. The primary objective of these mucosal vaccines would be to reduce the viral load in the nasopharyngeal cavity, likely in combination with existing approaches.

Alternatively, eliciting high sustained neutralizing IgG titers—higher than those currently achieved with the marketed vaccines—may give systemic vaccines sufficient capacity of mucosal protection against viral colonization at the entry site. There is still the possibility that recurrent boosts of mRNA vaccines, potentially adapted to the latest variant, may also achieve this goal. Otherwise, upcoming subunit vaccines based on the purified S protein alone or in combination with other viral proteins and a strong adjuvant may fulfill this specification. There is an urgent unmet need for comparative studies to assess these options.

Let us finish with a dream: what if the massive current attack rate of Omicron, a variant particularly fit in replication in the upper respiratory tract, led to natural mucosal immunity, similar to a mucosal vaccine? In synergy with the current high vaccine coverage at least in high‐income regions, would this suffice to offer disease elimination by reaching, at last, the elusive “Holy Grail” of herd immunity?
